# Electronic Noses: From Gas-Sensitive Components and Practical Applications to Data Processing

**DOI:** 10.3390/s24154806

**Published:** 2024-07-24

**Authors:** Zhenyu Zhai, Yaqian Liu, Congju Li, Defa Wang, Hai Wu

**Affiliations:** 1 National Institute of Metrology of China, Beijing 100029, China; zhaizyu@163.com (Z.Z.); wangdf@nim.ac.cn (D.W.); 2 Inner Mongolia Institute of Metrology Testing and Research, Hohhot 010020, China; 3 College of Textiles, Donghua University, Shanghai 201620, China; congjuli@126.com

**Keywords:** electronic nose, gas sensors, metal oxides, drift compensation

## Abstract

Artificial olfaction, also known as an electronic nose, is a gas identification device that replicates the human olfactory organ. This system integrates sensor arrays to detect gases, data acquisition for signal processing, and data analysis for precise identification, enabling it to assess gases both qualitatively and quantitatively in complex settings. This article provides a brief overview of the research progress in electronic nose technology, which is divided into three main elements, focusing on gas-sensitive materials, electronic nose applications, and data analysis methods. Furthermore, the review explores both traditional MOS materials and the newer porous materials like MOFs for gas sensors, summarizing the applications of electronic noses across diverse fields including disease diagnosis, environmental monitoring, food safety, and agricultural production. Additionally, it covers electronic nose pattern recognition and signal drift suppression algorithms. Ultimately, the summary identifies challenges faced by current systems and offers innovative solutions for future advancements. Overall, this endeavor forges a solid foundation and establishes a conceptual framework for ongoing research in the field.

## 1. Introduction

**Figure 1:**
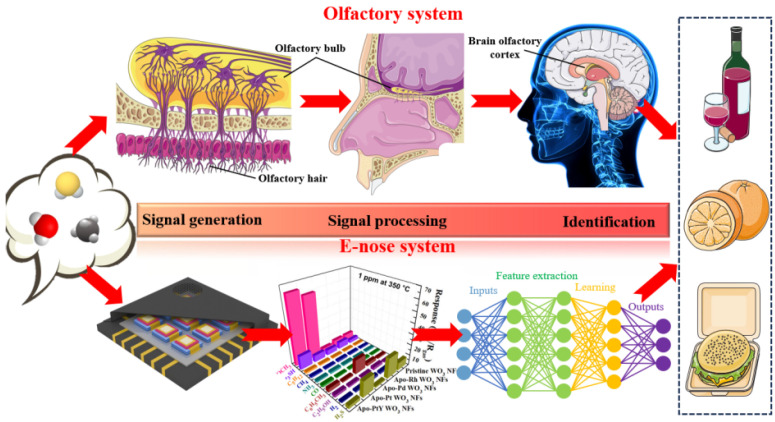
Schematic diagram of human olfaction and e-Nose.

The e-Nose is an electronic device designed to sense and analyze odors. It functionally and structurally mimics the olfactory system of humans as well as other mammals through a bionic approach. The process of gas recognition using the e-Nose system is compared with the human olfactory system, as shown in Figure 1. As early as 1964, Wilkens and Hartman pioneered the concept of electronic noses, simulating the olfactory process with electrodes that reacted chemically with gases. This marked the beginning of extensive research in the field. The commercialization of gas sensors took a significant step forward in 1967 with Japan’s Figaro company introducing the first metal oxide semiconductor (SnO_2_) gas sensors to the market. In 1982, Persaud et al. laid the groundwork for electronic noses by introducing a platform that included a gas sensor array and pattern recognition algorithms, enabling the analysis of mixed gases. In August 1991, the North Atlantic Treaty Research Organization convened its inaugural specialized conference on electronic noses in Iceland. Subsequently, research on electronic noses has proceeded at a rapid pace. “e-Nose” was first formally used in 1994 by Gardner, who subsequently co-sponsored the concept of the e-Nose with Bartlett [1]. An electronic nose is a sophisticated device that comprises an array of electronic chemical sensors with partial specificity and an appropriate pattern recognition system. This combination allows the electronic nose to recognize simple or complex odors [2]. In 1994, the world’s inaugural commercial electronic nose was launched.

**Figure 2:**
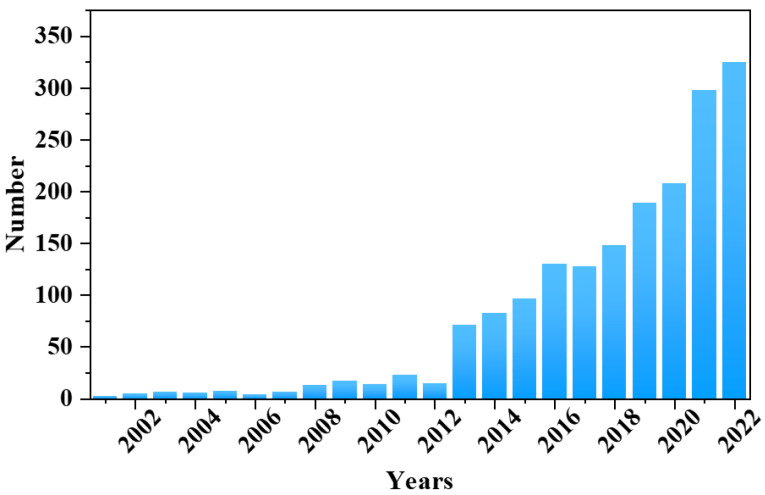
Number of articles on “e-Nose” in “Web of Science” per year.

Figure 2 shows the number of articles published per year by searching for “e-Nose” in the “Web of Science” website. Between 2000 and 2022, the number of published articles increased from 1 to 325, especially in recent years. As indicated by the data, with the continuous evolution and intersection of material science and artificial intelligence, the demand for advanced sensors in multiple industries is on the rise. This surge in demand is prompting an increasing number of researchers to explore the field of e-Noses more deeply.

**Figure 3:**
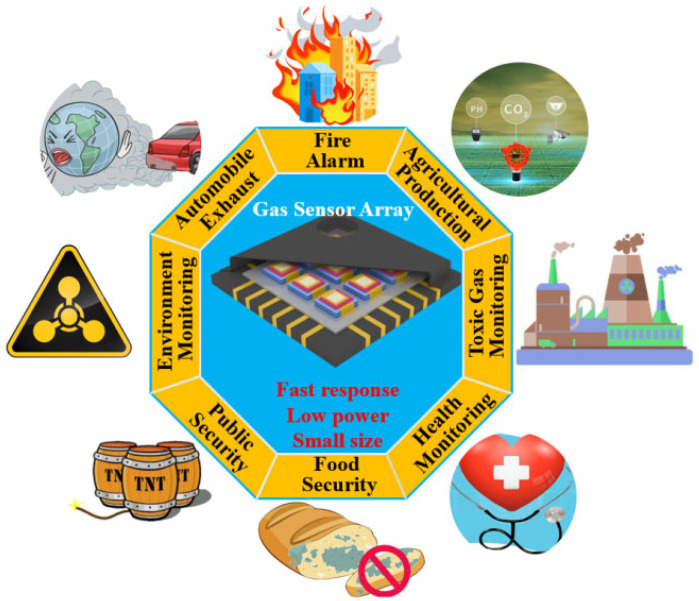
Application areas of e-Nose.

Qualitative and quantitative detection of multi-component gases has a wide range of applications in the fields of environmental control, food inspection, aerospace, medical monitoring, etc., as shown in Figure 3 [3]. Detecting multi-component gases with gas analyzers consisting of gas sensor arrays boasts the advantages of low cost, short measurement cycles, and real-time and on-line detection. However, the generally poor selectivity of gas sensors makes it difficult to realize the detection and analysis of multi-component gases using a single sensor. Currently, the most effective method is to form a sensing array of gas sensors with different detection properties and combine the response values into a sensing signal with multiple dimensions. Grounded in specific recognition algorithms, the type and concentration of the target gas can be more accurately detected.

With rapid advances in materials science and artificial intelligence, e-Nose technology needs to be modernized correspondingly. Improvements are mainly made from two aspects: The first is the modification of the test system, most notably the optimization of the gas-sensitive material and sensor structure. Specifically, efforts should be made to reduce the number of sensors in the sensing array by improving the selectivity of the gas-sensitive material to enhance the detection efficiency. In addition, in terms of signal processing techniques, appropriate data analysis methods should be chosen to improve the robustness of the system.

Gas sensors, as the core components of the e-Nose, include electrochemical gas sensors, semiconductor gas sensors, catalytic combustion gas sensors, and solid electrolyte gas sensors [4,5]. At present, the Metal Oxide Semiconductor (MOS) stands out as the most popular and technologically mature class of gas sensors, featuring advantages of low cost, high sensitivity, fast response time, and simple structure, such as SnO_2_, ZnO, and Fe_2_O_3_ [6]. However, MOS sensors generally have poor selectivity, and are currently mainly employed to enhance sensor performance by compositing and doping with nanomaterials. Advancements in the development and fabrication of MOS materials are set to broaden their application horizons [7].

Metal-Organic Frameworks (MOFs) serve as porous nanomaterials, characterized by a wealth of metal active sites and functional groups on their surfaces. These features make MOFs well-suited for enhancing surface adsorption and facilitating reactions with specific gas molecules [8]. Therefore, MOF materials have been gradually adopted as gas-sensitive materials for gas sensors, forming specific adsorption with the target gas through the ligand bonds in the framework. After the target molecules are enriched inside the backbone of the material, the physical or chemical properties of the material will be changed. Subsequently, the sensor converts this alteration into an electrical signal for the qualitative and quantitative detection of the target gas. The advantage of MOFs over MOS materials lies in that MOFs-based gas sensor can detect gases at room temperature, effectively reducing sensor drift caused by temperature changes [9]. Therefore, MOFs and MOS materials have complementary properties, and the combination of these two is expected to be a new direction for the subsequent development of gas sensors.

The signal output from the sensing array needs to be analyzed by adopting corresponding algorithms, and the common steps involve signal preprocessing and feature extraction, classification, and regression. The curve must first undergo preprocessing, including baseline compensation and amplitude normalization, to eliminate low-amplitude baseline drifts from the detected signal. Subsequently, by extracting the features, the extracted features are classified and regressed according to the appropriate algorithms. To characterize the gas, common classification methods in the literature include K-Nearest Neighbor (K-NN), Artificial Neural Network (ANN), and Support Vector Machine (SVM). If the analysis purpose is to quantify the gas, regression algorithms such as Partial Least Squares Regression (PLSR), ANN, Support Vector Regression (SVR), etc. can be adopted.

In practical applications, drift is one of the main difficult problems faced by the e-Nose in the measurement process [10]. Specifically, sensors of the same model or even from the same batch are not guaranteed to have identical sensitivity characteristics, resulting in instrumental variation in the e-Nose. In addition, prolonged use of the gas-sensitive material can result in aging, poisoning, and corrosion, jointly leading to time-varying-drift. Humidity and temperature disturbances in the test environment can also cause the e-Nose to drift [11,12]. Therefore, the drift problem has become a major obstacle that restricts the practical application and promotion of e-Nose, and has become a “short-board” problem.

In this paper, the gas-sensitive materials, application areas, and data processing algorithms for the e-Nose are presented. In the first part, ZnO, SnO_2_, Fe_2_O_3_, and MOFs are mainly introduced in terms of gas sensor components; in the second part, e-Noses are summarized in terms of applications in disease diagnosis, environmental monitoring, agriculture, and food safety detection; and the third part summarizes data processing methods commonly used in the e-Nose system, including pattern recognition as well as signal drift suppression algorithms. Finally, ideas for further development of an e-Nose based on the current study are proposed and discussed to provide a basis for subsequent research.

## 2. Sensors in the e-Nose System

### 2.1. MOS-Based Gas Sensors

**Figure 4:**
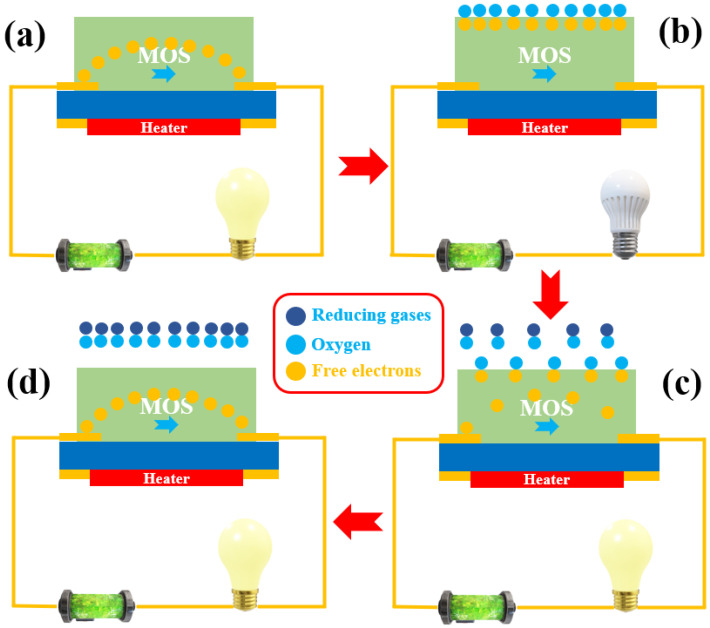
(**a**) Movement of free electrons to achieve N-type semiconductor conductivity; (**b**) Oxygen in the air adsorbs free electrons in N-type semiconductor; (**c**) Reducing gases react with adsorbed oxygen on the N-type semiconductor surface, resulting in the release of some free electrons; (**d**) The electrons flow back to the semiconductor, resulting in a decrease in the concentration of holes and an increase in resistance.

MOS, as an ideal gas-sensitive material, has seen extensive applications in gas detection. Among the most commonly used metal oxides are SnO_2_, ZnO, Fe_2_O_3_, and WO_3_ [13]. MOS materials are inherently classified as either N-type or P-type. In the case of N-type MOSs, the conduction band of the semiconductor is populated with a substantial number of free electrons when in the presence of air. Upon exposure to ambient air, the surface of the N-type metal-oxide semiconductor rapidly adsorbs oxygen molecules from the atmosphere. The free electrons in the MOS are captured by oxygen molecules, forming strongly reactive oxygen anions (O_2_^−^, O^−^, O^2−^) under different temperature conditions. As the number of free electrons inside the MOS decreases, the resistance value shows an increase [14]. When reducing gases interact with the adsorbed oxygen, the captured free electrons can be released back into the MOS, resulting in a decrease in resistance. As the concentration of the reducing gas increases, more free electrons are released, and thus the magnitude of the resistance of the MOS is usually inversely related to the value of the reducing gas concentration. The schematic diagram is shown in Figure 4.

P-type semiconductors are semiconductor materials in which the majority of carriers are holes and the minority of carriers are electrons. Since the holes are positively charged, under the action of an applied electric field, the holes move in the same direction as the electric field, giving P-type semiconductors unique conductive properties. When the P-type semiconductor gas sensor is exposed to the gas to be measured, gas molecules adsorb, desorb, or chemically react with the surface of the semiconductor material. These reactions affect the state of charge on the semiconductor surface and thus change the conductivity of the semiconductor. When the gas to be measured is a reducing gas, it will react with the oxygen ions on the surface of the semiconductor, releasing electrons back into the semiconductor, resulting in a decrease in the concentration of holes and an increase in resistance; conversely, when the gas to be measured is an oxidizing gas, it will capture electrons in the semiconductor, resulting in an increase in the concentration of holes and a decrease in resistance.

#### 2.1.1. ZnO

ZnO, an n-type semiconductor with a band gap of 3.37 eV, is renowned for its exceptional gas sensitivity. Its high electron mobility and robust chemical and thermal stability make it an outstanding material for gas-sensitive applications [15]. However, the poor selectivity, high detection limits, and high operating temperatures hinder its development [16]. The limit of detection (LOD) of ZnO as a gas sensitive material for gas sensors as well as the test temperatures are summarized in Table 1. ZnO gas sensors have excellent response not only for NO_2_ and H_2_S, but also for CO, O_2_, NH_3_, and some VOCs (Triethylamine, Acetone). ZnO gas sensors mainly detect target gases by measuring the change in electrical resistance after exposure to the target gas. Taking NO_2_ gas as an example, when ZnO is exposed to oxidizing gas (NO_2_), the gas molecules react with the adsorbed oxygen on the surface of ZnO due to the higher electron affinity energy of the NO_2_ gas. This results in a decrease in the electron density and an increase in resistance. As the concentration of NO_2_ gas increases, more adsorbed oxygen on the surface of ZnO reacts with the NO_2_ gas molecules, and the resistance of the sensor decreases linearly with NO_2_ gas concentration [17].

According to Table 1, the main problem with MOS gas sensors is that they have optimal efficiency at high temperatures (150–400 °C), leading to high energy consumption and bringing about risks such as gas explosions. As a result, a series of material engineering techniques have been developed to reduce the testing temperature of the sensors, including metal doping, surface functionalization, and construction of heterojunctions and nanomaterial composites. Among them, ultraviolet (UV) and visible light irradiation technologies are gaining increasing attention as simple, fast, and effective methods [18]. 

**Table 1:** Summary of representative ZnO gas sensors.

Sensing Materials	Temperature (°C)	Gas	Type	LOD (ppm)	Ref.
ZnO-NPs/MEMS	RT	NH_3_	Resistance	0.1	[19]
ZnO nanosheet	300	CO	Resistance	0.134	[16]
NiO-ZnO	RT	Acetone	Resistance	10	[20]
CuO-ZnO	225	H_2_S	Resistance	2	[21]
CuO-ZnO	100	H_2_S	Resistance	100	[22]
CuO/ZnO nanorods	500	H_2_S	Resistance	50	[23]
CeO_2_/ZnO	120	NO_2_	Resistance	0.1	[24]
	25	NO_2_	Resistance	0.5	
ZnO nanowire	60	Ethanol	Resistance	50	[25]
ZnO nanowire	300	CO	Resistance	0.139	[17]
	350	NO_2_	Resistance	0.1	
CuO/ZnO	250	NO_2_	Resistance	1	[26]
α-Fe_2_O_3_/ZnO	250	H_2_S	Resistance	1	[27]
CdO-ZnO	350	Formaldehyde	Resistance	50	[28]
Porous-ZnO	200	NO_2_	Resistance	0.5	[29]
ZnO/CuO nanorods	50	H_2_S	Resistance	1	[30]
ZnO/MoO_3_	180	Triethylamine	Resistance	0.1	[31]
Pt-ZnO	200	Triethylamine	Resistance	8	[32]
Pt_1_-ZnO	200	Triethyamine	Resistance	0.1	[33]
Pd-ZnO	280	Aniline	Resistance	0.5	[34]
Al-ZnO	200	O_2_	Resistance	25,000 (2.5%)	[35]

**Figure 5:**
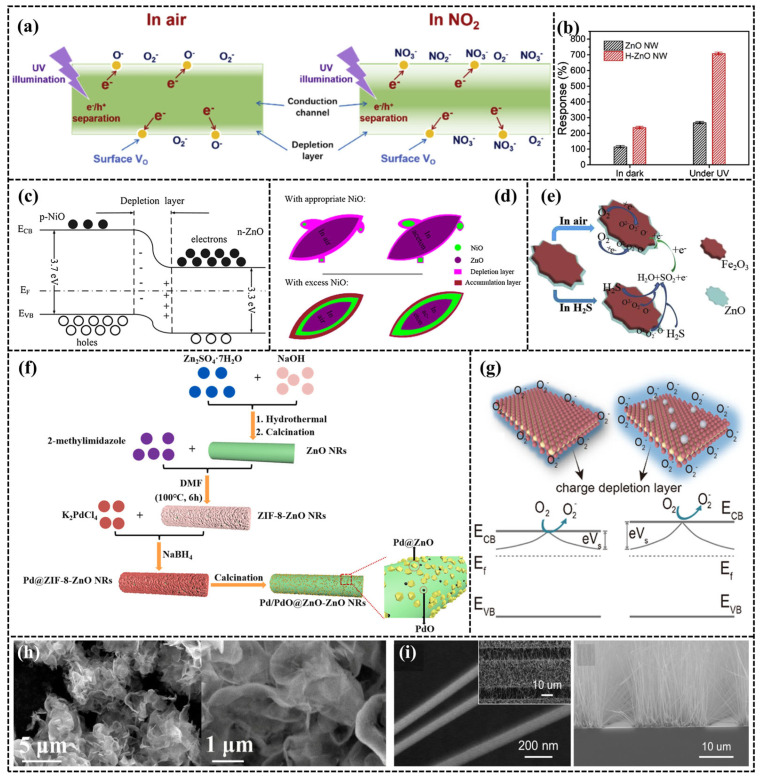
(**a**) Schematic illustration of the mechanism for synergistic effects of UV activation; (**b**) NO_2_ gas sensing performance of ZnO nanowires [38]; (**c**) Schematic representation of band configuration at the interface of the NiO/ZnO nanoheterostructure; (**d**) Schematic gas sensing mechanism with different amounts of NiO; (**e**) Schematic illustration of sensing mechanism based on ZnO/Fe_2_O_3_ [20,22,27]; (**f**) Schematic illustration for the synthesis of Pd/PdO@ZnO-ZnO NRs [32]; (**g**) Band structure of ZnO and Pt_1_/ZnO [33]; (**h**) Low and high-magnification FESEM images of ZnO; (**i**) Cross-sectional FE-SEM images of ZnO NWs [34].

Many hole–electron pairs are induced in the MOS by UV irradiation, which causes the excited electrons to jump from the valence band to the conduction band, significantly reducing the depletion layer in the MOS [36,37]. When irradiated with UV irradiation with photon energy greater than the width of the forbidden band of the MOS, electron–hole pairs are generated inside the MOS, in which holes move to the surface of the MOS under the action of the built-in electric field on the surface, combine with O_2_^−^ on the surface, and desorb the adsorbed oxygen, leaving the unpaired electrons that have been excited in the conduction band. Therefore, the current of MOS devices under UV irradiation becomes large. However, when the UV light is continuously irradiated, the current of the device decreases again, which is mainly due to the fact that the photogenerated electrons combine with oxygen to form O_2_^−^ _(*hv*)_, which reduces the electron concentration in the MOS. Concomitantly, electrons and holes modify the state of the defects in the MOS, thereby influencing the adsorption capacity of the semiconductor surface. Light can facilitate the response and recovery of the gas, which can be explained by the fact that photoexcitation can enhance the rate of adsorption and desorption of the gas. Specifically, Wang et al. constructed a layer of ZnO as the gas-sensitive layer on the thin film material utilizing the in situ growth method. The sensor shows excellent response strength and response/recovery speed for NO_2_ gas under room-temperature (RT) UV conditions, with a minimum detection concentration of 20 ppb [38]. The reason lies in the fact that ZnO can decompose more free electrons under the irradiation (especially UV), which enhances the electrical conductivity of ZnO, as shown in Figure 5a,b. Other optimization methods include modification and doping.

To enhance the sensitivity of ZnO-based gas sensors, two main modification strategies are employed. The first involves creating heterojunctions with other semiconductors. Due to the different Fermi energy levels between the MOSs, the flow of electrons leads to the formation of a p–n junction between the two MOS materials, which puts the material in a highly resistive state. After the target molecule reacts with adsorbed oxygen, the p–n junction converts into a Schottky barrier, causing a rapid electron transfer between the two MOSs and a subsequent significant decrease in the resistance value. NiO, CuO, and Fe_2_O_3_ are commonly used to form heterojunctions with ZnO. For homogeneous heterojunctions (n–n, p–p), they have similar sensing mechanisms [20,22,27]. Figure 5c–e present the schematic diagrams of the sensing mechanisms of NiO/ZnO and Fe_2_O_3_/ZnO.

The alternative approach involves enhancing the gas-sensitive properties of ZnO by doping with precious or non-precious metals. The working mechanism of precious metals (e.g., Pt, Au, Ag) doped with ZnO can be understood as electron sensitization. The catalytic reaction of the gas molecules on the surface of noble metals accelerates the reaction with the adsorbed oxygen, thereby enhancing the performance of the sensor [32]. The synthesis roadmap of Pd@ZnO NRs is shown in Figure 5f. Non-precious metals (e.g., Al, Cu) enhance the sensor performance by changing the band gap, electronic structure, and surface activity of the MOS [35]. Advancements in catalyst development and characterization technologies have revealed that reducing nanocrystal size to atomic clusters or single atoms leads to fundamental shifts in energy level and electronic structures. Thus, compared to conventional nanomaterials, MOS doped with single atoms exhibits better response strength, stability, and selectivity in gas sensors. For instance, Liu et al. [33] concluded that the response strength and response/recovery time of Pt_1_/ZnO containing Pt single atoms to triethylamine (TEA) were significantly enhanced compared to pure ZnO and Pt nanoparticles (NP)/ZnO. The oxygen vacancies in Pt_1_/ZnO promote oxygen adsorption and activation, while the formation of Pt–N bonds between Pt single atoms and N in the TEA molecule promotes the rapid adsorption, as shown in Figure 5g.

The morphological structure of the gas-sensitive material also has a significant effect on the performance of the sensor, especially the microstructure. The common nano-morphologies of ZnO in the literature are nano-sheets, nano-rods, nano-wires, nano-cones, nano-plates, nano-flowers, etc. The main purpose is to increase the specific surface area of the ZnO structure, thereby offering an increased number of adsorption and reaction sites to boost the efficacy of gas sensors [16,23,25]. ZnO can be made porous through methods such as solvent heating, which increases the diffusion rate of gas molecules and enlarges the contact area, thereby boosting the gas sensor’s performance. Zhang et al. [34] observed that ZnO nano-cones with higher specific surface area presented better sensing performance for NO_2_ gas compared to ZnO nano-plates and ZnO nano-flowers. The microstructures of ultrathin agaric-like ZnO@Pd and ZnO nano-wires are compared in Figure 5h,i.

#### 2.1.2. SnO_2_

SnO_2_, as a traditional MOS material, has been extensively used for monitoring combustible gases such as methane since the 1970s. The gas-sensing mechanism of SnO_2_ is the same as that of most MOS materials and is mainly based on gas adsorption–desorption as well as ambient oxygen exchange, which can be explained using the Schottky barrier model theory [39,40]. Therefore, SnO_2_ is often used in e-Nose systems. Representative applications of SnO_2_ gas sensors are summarized in Table 2.

**Figure 6:**
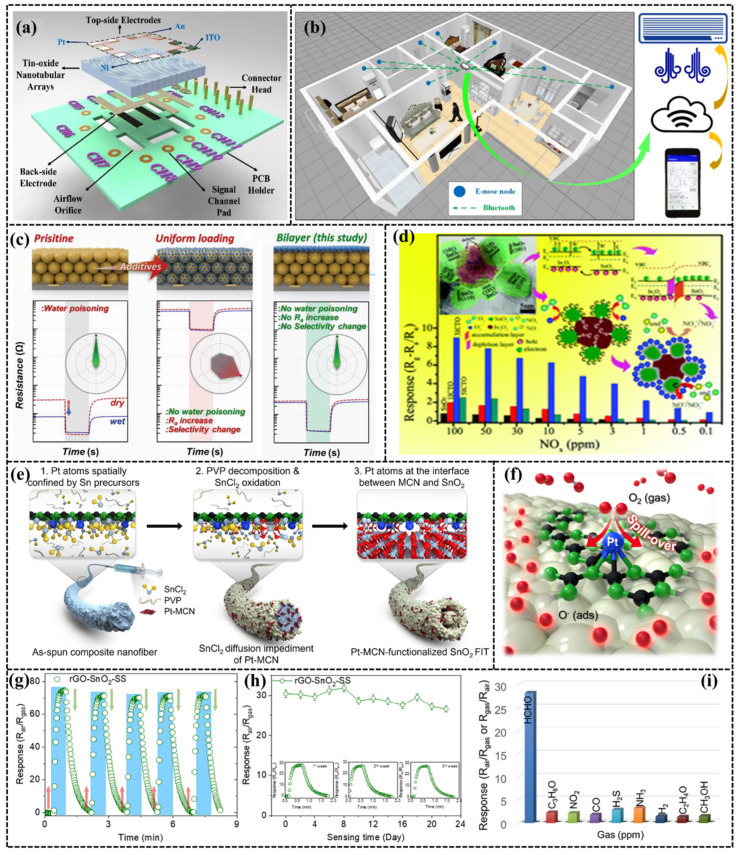
(**a**) e-Nose packaging structure; (**b**) Illustration of the proposed e-Nose application in future smart buildings [42]; (**c**) Water poisoning behavior of pristine oxide sensors [51]; (**d**) Schematic diagram of sensing performance and mechanism of In_2_O_3_/SnO_2_ applied to NO_x_ [52]; (**e**) Schematic illustration of the Pt SA delivery process and nanofiber-in-tube structure formation; (**f**) Schematic illustration of O_2_ dissociation over Pt SAs on Pt-MCN-SnO_2_ and chemisorbed oxygen species [43]; (**g**) Sensing response characteristics of the fabricated rGO-SnO_2_-SS sensor; (**h**) Long-term stability of the rGO-SnO_2_-SS sensor; (**i**) Selectivity of the fabricated rGO-SnO_2_-SS sensor [45].

From a summary of the literature, it can be concluded that SnO_2_-based gas sensors are basically resistive and have superior response performance for NO_x_ gases. At the same time, H_2_S, H_2_, and some VOCs exhibit certain response strength. SnO_2_ gas sensors, like ZnO, also exhibit optimal operating efficiencies at high temperatures (150–300 °C) and can be detected at room temperature under UV irradiation [41]. Consequently, incorporating a heating or UV unit into the sensor design complicates the equipment and increases energy consumption. Chen et al. [42] designed an SnO_2_ gas sensor with a nanotube array structure, which detects H_2_, NO_2_, and benzene gases at room temperature. This innovative device is Bluetooth-enabled, allowing it to connect to a smartphone. Users can monitor the presence of target gases in real-time via an app. The schematic diagrams of the sensor structure and detection system are shown in Figure 6a,b, respectively.

**Table 2:** Summary of representative SnO_2_ gas sensors.

Sensing Materials	Temperature (°C)	Gas	Type	LOD (ppm)	Ref.
Pt- carbon nitride/SnO_2_	275	Formaldehyde	Resistance	0.05	[43]
F-SnO_2_ hollow fibers	RT	Ethanol	Resistance	10	[44]
CNTs/SnO_2_	RT-UV	NO_2_	Resistance	0.02	[41]
rGO-SnO_2_	100	Formaldehyde	Resistance	0.01	[45]
SnO_2_-Ti_3_C_2_T_x_	150	NO_2_	Resistance	24.8	[46]
ZnO quantum dots@SnO_2_	225	Formaldehyde	Resistance	0.005	[47]
SnO_2_/ZnO	275	Ethanol	Resistance	18.1	[48]
SnO_2_ nanoflower	RT-UV	NO_2_	Resistance	0.2	[49]
Sb-SnO_2_	40	H_2_S	Resistance	0.004	[50]
Pt-SnO_2_ nanotube	RT	H_2_	Resistance	50	[42]
		NO_2_	Resistance	5	
		Benzene	Resistance	2	
Tb_4_O_7_-SnO_2_	350–450	Acetone	Resistance	-	[51]
In_2_O_3_-SnO_2_	RT	NO_x_	Resistance	0.1	[52]
WO_3_-SnO_2_	110	Methane	Resistance	10	[53]
SnO_2_-6 squama-wrapped tubes	92	NO_2_	Resistance	0.1	[54]
	170	Diisopropylamine	Resistance	1	

Water vapor acts as an inevitable and strong interfering gas for oxide semiconductor gas sensors. Thus, the water poisoning of sensors has been a major impediment to reliable gas sensing during the past six decades [51,55]. Water molecules usually deplete the active sites on the MOS surface, leading to a decrease in the sensor’s response performance for the target gas [51].

To avoid the influence of water molecules on performance, additives, and dopants (e.g., Pd, Sb, NiO, CuO, Tb, etc.) are usually added to the gas-sensitive material [56,57,58]. For instance, Jeong et al. [51] improved the humidity stability by integrating a hydrophobic Tb_4_O_7_ layer as a protective layer on the MOS surface in situ. In addition, they experimentally demonstrated the strong immunity of the composite with different MOS (In_2_O_3_, SnO_2_) materials to humidity variations, as shown in Figure 6c. The double-layer structure of gas-sensitive materials is widely applicable and does not affect the performance of the original materials.

Furthermore, SnO_2_ can be compounded with other MOSs (such as ZnO, WO_3_, In_2_O_3_) to form heterojunctions and to enhance the performance of gas sensors [48,52,53]. Xu et al. [52] prepared In_2_O_3_@SnO_2_ nanorods by electrostatic spinning followed by calcination, and found that In_2_O_3_ could effectively improve the carrier concentration and oxygen vacancies in SnO_2_. In particular, the sensor exhibits excellent gas-sensitive response to NOx at room temperature when Sn/In = 25:0.3. The highest response value of the In_2_O_3_@SnO_2_ for NOx (100 ppm) is more than 11 times that of pristine SnO_2_ nanorods at room temperature, and the LOD can be 0.1 ppm, as shown in Figure 6d. After compounding WO_3_ with SnO_2_, the response of the WO_3_-SnO_2_-based sensor to 500 ppm methane at 110 °C is 2.3 times of that of pure SnO_2_-based sensors [53].

Enhancing the efficiency of a catalyst often involves reducing it to a single-atom form [59]. Utilizing single-atom catalysts (SACs) in catalysis maximizes atom utilization efficiency, especially as the scale of the catalysts is reduced. Consequently, MOS-based gas sensors that incorporate single-atom doping demonstrate exceptional response characteristics and selectivity. Compositing single atoms with SnO_2_ has also become a common method to enhance the response performance of sensing, in which Pt single atoms, as an efficient and mature catalyst, can be significantly enhanced after doping with SnO_2_. Shin et al. [43] trapped the single atoms (Pt) at heterojunctions of a carbon nitride/SnO_2_ heterostructure. The detection limits for H_2_, NO_2_, benzene, and other gases are low at room temperature, and the LOD for formaldehyde reaches 50 ppb under heated conditions (275 °C), as shown in Figure 6e,f.

Forming heterojunctions of SnO_2_ with MOS or doping single atoms such as Pt can significantly enhance the sensing performance for specific gases. However, these methods typically require operation at elevated temperatures, which can considerably reduce the sensor’s operational lifespan [42,47,48]. The preparation and modification of carbon-based materials have consistently garnered significant interest. Graphene, in particular, is frequently utilized for the composition and modification of materials due to its special physicochemical properties. Considering the advantages of graphene’s narrower band gap, rapid carrier migration, and high specific surface area, the composites prepared are widely used in different fields, especially graphene oxide (GO) and reduced graphene oxide (rGO). The surface of GO is rich in hydrophilic functional groups (hydroxyl and carboxyl groups), and rGO with excellent chemical stability is obtained by removing some of the surface functional groups and restoring the π-bonds through reductive treatment. Lee et al. [45] doped 3D-rGO into SnO_2_ nanospheres (rGO-SnO_2_-SS) to detect formaldehyde in breath gas, and the LOD could reach 10 ppb at a relatively low temperature (100 °C). The performance of the sensor was also tested, as shown in Figure 6g–i.

#### 2.1.3. Fe_2_O_3_

**Figure 7:**
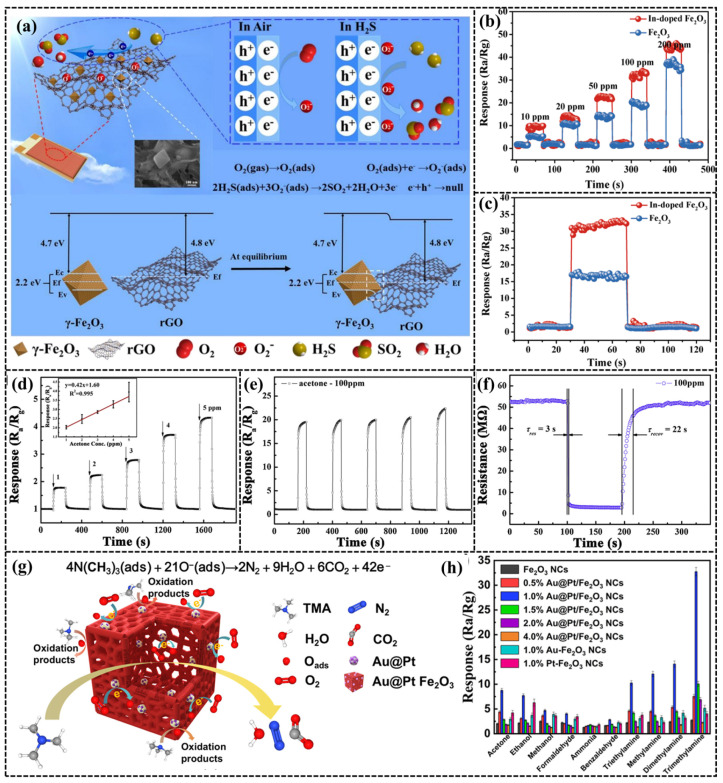
(**a**) Schematic for the mechanism of H_2_S sensing of γ-Fe_2_O_3_/rGO composites [62]; (**b**) Concentration-dependent response curves; (**c**) Dynamic response–recovery curve; (**d**) Pt-Fe_2_O_3_ when subjected to low concentrations of acetone at 139 °C [65]; (**e**) Cyclability of the sensor based on Pt-Fe_2_O_3_ toward 100 ppm of acetone at 139 °C; (**f**) Response and recovery time of Pt-Fe_2_O_3_ to 100 ppm of acetone at 139 °C [69]; (**g**) Schematic illustration of the TMA gas-sensing mechanism; (**h**) Selectivity of Au@Pt/Fe_2_O_3_ type gas sensors [71].

Fe_2_O_3_-based gas sensors are characterized by excellent response performance, low cost, and wide applicability. In Table 3, it can be seen that Fe_2_O_3_ boasts high selectivity mainly for H_2_S, acetone, NO_2_, and some VOCs, and the optimal testing temperature is between 100 and 300 °C. This indicates that Fe_2_O_3_ is subject to the same disadvantages of high operating temperature and poor selectivity as ZnO and SnO_2_. It can also be complexed with rGO to enhance the response performance of the sensor and to adsorb the target gas using the active sites on the rGO surface [60]. However, rGO is susceptible to aggregation, which can hinder the performance of composite materials [61]. Therefore, porous MOF materials can be taken as precursors, and rGO can be uniformly dispersed in Fe_2_O_3_ to expose more active sites. Zhang et al. [62] adopted Fe-MOFs (MIL-88) as the precursor of Fe_2_O_3_, dispersed rGO homogeneously within MIL-88, and calcined to obtain rGO/Fe_2_O_3_. The sensor has excellent response performance for H_2_S gas at room temperature, and the LOD can reach 0.39 ppm. The schematic diagram is shown in Figure 7a.

**Table 3:** Summary of the representative of Fe_2_O_3_-gas sensor.

Sensing Materials	Temperature (°C)	Gas	Type	LOD (ppm)	Ref.
γ-Fe_2_O_3_/rGO	RT	H_2_S	Resistance	0.39	[62]
α-Fe_2_O_3_	RT	NO_2_	Resistance	1	[63]
α-Fe_2_O_3_/ZnO	250	H_2_S	Resistance	1	[27]
α-Fe_2_O_3_/graphene	280	Ethanol	Resistance	1	[64]
In-Fe_2_O_3_	320	Acetone	Resistance	31.7	[65]
Fe_2_O_3_/In_2_O_3_	200	Acetone	Resistance	10	[66]
α-Fe_2_O_3_	150	Ethanol	Resistance	50	[67]
Pt-α-Fe_2_O_3_	375	Dimethyl disulfide	Resistance	5	[68]
Pt-Fe_2_O_3_	139	Acetone	Resistance	0.2	[69]
Mn-α-Fe_2_O_3_	300	H_2_	Resistance	10	[70]
Au@Pt/α-Fe_2_O_3_	150	Trimethylamine	Resistance	1	[71]
Fe_2_O_3_ (Hierarchical and Hollow)	50	H_2_S	Resistance	0.25	[72]
α-Fe_2_O_3_@ZnO@ZIF-8	200	H_2_S	Resistance	0.2	[73]

Doping particles with catalytic properties into the Fe_2_O_3_ structure using the hydrothermal methods is also one of the ways to enhance the performance of the sensor. Common catalysts used in this process include In, among others. Zhang et al. [65] doped In^3+^ into the Fe_2_O_3_ structure, significantly improving the response to acetone. The response/recovery time is 1 s. The response curves before and after doping with In^3+^ are compared in Figure 7b,c. The doping of In^3+^ reduces the lattice size of Fe_2_O_3_, enhances the specific surface area, and exposes more active sites [74]. However, compared to Fe_2_O_3_/In_2_O_3_ with a heterogeneous structure, the LOD for acetone at 200 °C can reach 10 ppm [66]. Fe_2_O_3_/In_2_O_3_ can detect lower acetone concentrations at lower temperatures. This enhanced capability is attributed to two factors: firstly, the incorporation of Fe_2_O_3_ increases the number of active sites, and secondly, the heterojunction formed between In_2_O_3_ and Fe_2_O_3_ improves the gas-sensitive properties of the sensor.

Compared to heterojunctions, Pt, as a traditional and effective catalyst, continues to offer considerable benefits when applied to gas-sensitive materials. For instance, Zhang et al. [69] doped Pt with Fe_2_O_3_, and found that the LOD could achieve 0.2 ppm for acetone. The mechanism of sensing performance enhancement can be explained by the excellent chemical and electronic sensitization of Pt nanoparticles and their oxidized state, as shown in Figure 7d–f. However, the selectivity of the Pt-Fe_2_O_3_ sensor is still insufficient, which is reflected in the strong response of the sensor not only to acetone gas but also to dimethyl disulfide [68]. Shen et al. [71] enhanced the response and selectivity of the sensor for trimethylamine utilizing bimetallic nanocrystals doped with α-Fe_2_O_3_. The limit of detection for trimethylamine was achieved at 1 ppm at a lower temperature of 150 °C, which was employed to determine the freshness of fish. Details are presented in Figure 7g,h.

Efforts have also been made to enhance the selectivity and sensitivity of gas sensors based on Fe_2_O_3_ or other MOS materials. These endeavors involve loading with noble metals, doping with catalytic oxides, and composing p–n junctions. However, it should be noted that the sensor may face some challenges with high LOD and operating temperature. To address this, improving the material’s specific surface area through the modification of Fe_2_O_3_’s pore-like structure could be beneficial. For instance, designing it as a hollow structure could potentially improve its performance [72]. Another possibility is to increase the selectivity and adsorption of the target gas by combining Fe_2_O_3_ with other porous materials (such as MOFs). This combination has the potential to further improve the noise immunity and LOD of the sensor [73].

### 2.2. MOF-Based Gas Sensors

The MOSs mentioned above (SnO_2_, ZnO_2_, Fe_3_O_4_) are advantageous due to their low cost, ease of processing, and high performance. However, they also have some drawbacks, such as high operating temperatures (200–400 °C) and poor selectivity. These limitations highlight the need for integrating additional sensors and implementing temperature control systems in e-Nose arrays to ensure reliable operation of these materials [75]. The rapid development of nanomaterials has spurred an increase in research focused on creating gas sensors with heightened sensitivity and selectivity. One such example is Metal-Organic Frameworks (MOFs), known as porous frameworks formed by coordinating metal ions/clusters with organic ligands. Due to their high specific surface area and numerous active sites, MOFs have been extensively researched in the fields of gas storage [76], sensing [77], separation [78], and catalysis [79].

**Figure 8:**
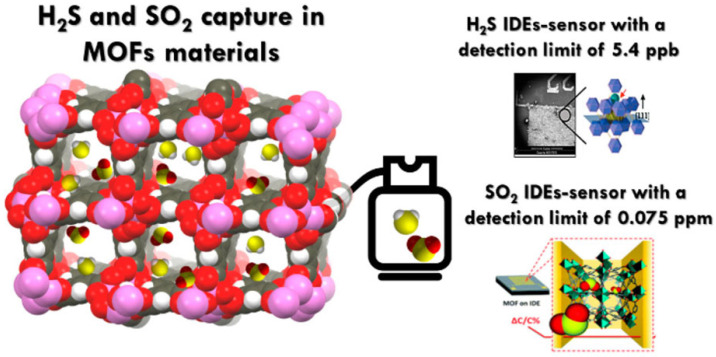
Schematic diagram of MOFs capacitive gas sensor mechanism [80].

Especially in the field of sensing, nanomaterials are not only utilized as precursors for crafting porous materials, but they can also be tailored to interact with target gases, thereby improving the sensor’s selectivity and sensitivity. When gas molecules enter the MOF frame, the physical or chemical properties of the material change accordingly. Subsequently, the sensor converts this change into an electrical signal for qualitative or quantitative detection of the target gas, as shown in Figure 8 [80]. MOF materials are thus applied as dielectric layer materials for capacitive gas sensors, and the research is mainly focused on the detection of gases such as H_2_S, SO_2_, CO_2_, NH_3_, VOCs gases, and humidity. Meanwhile, optimization methods to improve the stability, selectivity, and sensitivity of the sensors can be proposed based on the mechanism of gas adsorption by MOFs.

**Figure 9:**
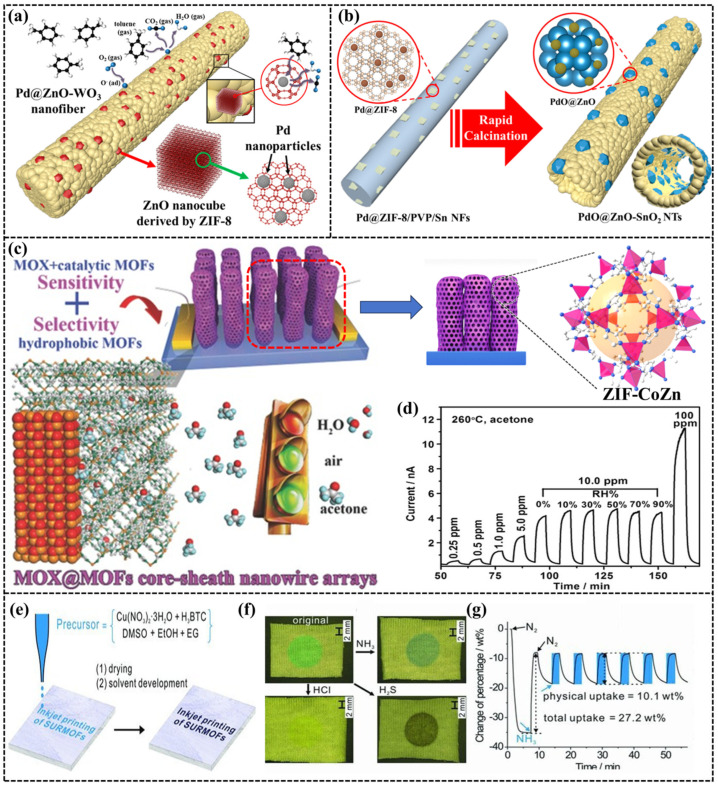
(**a**) Schematic illustration of toluene-sensing mechanism for Pd@ZnO–WO_3_ NFs [81]; (**b**) Schematic illustration of synthetic process of PdO@ZnO–SnO_2_ NTs [82]; (**c**) Schematic illustration of the preparation of ZnO@ZIF-CoZn gas sensors; (**d**)response–recovery curves of ZnO@5 nm ZIF-CoZn to acetone [94]; (**e**) Inkjet printing of SURMOFs onto flexible substrates using a HKUST-1 precursor solution as “ink”; (**f**) Photographs of a dot of HKUST-1 printed onto textile (5cy) before and after exposure to different gases; (**g**) Partially reversible adsorption/desorption of NH_3_ on HKUST-1 film [96].

Table 4 summarizes studies on the application of MOFs in gas sensing, predominantly in capacitive sensors, with a minority exploring their use in resistive or mass sensors. The primary reason is that most MOF materials have poor electrical conductivity, and the change in resistance value after interaction with the target molecule is not easy to detect. MOS in resistive sensors is primarily in combination with MOS materials or as a precursor to MOS materials. Koo et al. [81] used ZIF-8 with porous properties as the dispersant, combining Pd@ZnO-WO_3_ by electrostatic spinning with calcination, and as a resistive gas sensor for the detection of toluene. The uniform dispersion of Pd^2+^ in Pd@ZnO-WO_3_ by ZIF-8 can effectively improve the detection ability for the target gas, as shown in Figure 9a. Based on the research of Pd@ZnO-WO_3_, Koo et al. [82] replaced the fiber substrate material from WO_3_ to SnO_2_, and prepared the SnO2 nanofibers as a hollow tubular structure, further improving the sensitivity of the sensor by enhancing the contact area with the target gas, as shown in Figure 9b.

**Table 4:** MOFs-based gas sensors.

Gas	Sensing Materials	Temperature (°C)	Type	LOD (ppm)	Ref.
H_2_S	fum-fcu-MOF	RT	Capacitive	5.4 ppb	[83]
H_2_S	Ag_2_O@UiO-66-NO_2_	RT	Capacitive	1 ppm	[84]
CO_2_	NbOFFIVE-1-Ni	105	Capacitive	400 ppm	[85]
CO_2_	Mg-MOF-74	RT	Capacitive	200 ppm	[86]
SO_2_	MFM-300	RT	Capacitive	5 ppb	[87]
NH_3_	NDC-Y-fuc-MOF	RT	Capacitive	100 ppb	[88]
Methanol	NH_2_-MIL-53 (Al)	RT	Capacitive	-	[89]
Methanol	MIL-96 (Al)	-	Capacitive	-	[90]
Methanol	Cu-BTC	30	Capacitive	100 ppm	[91]
Methanol	Cu-BTC	25	Capacitive	47.3 ppm	[92]
Ethanol	Cu-BTC	25	Capacitive	150.5 ppm	[92]
H_2_O	Cu-BTC	RT	Capacitive	11.3 RH%	[93]
Toluene	Pd@ZnO-WO_3_ (ZIF-8)	350	Resistive	100 ppb	[81]
Acetone	Pd@ZnO-SnO_2_	400	Resistive	10 ppb	[82]
H_2_S	ZIF-8/ZnO	25	Resistive	50 ppb	[94]
Acetone	ZnO@ZIF-CoZn	260	Resistive	0.25 ppm	[95]
NH_3_	Cu-BTC	RT	Mass	-	[96]

The MOF materials serve solely as precursors for the MOS materials, with their active sites remaining underutilized. Wu et al. [94] and Yao et al. [95] proposed a shell-and-core structured MOF/MOS composite for use as gas-sensitive materials. In this composite, MOFs primarily function in selective adsorption and enrichment of the target gases, as shown in Figure 9c. In addition, the MOFs also can be used as a filter layer to filter out water molecules, as well as other impurity gases, to enhance the stability and selectivity under different humidities, as shown in Figure 9d. MOFs adsorb the target gas and then change in weight and color for sensing. Zhuang et al. [96] sprayed the MOFs onto the paper according to the designed pattern using printer inkjet technology, as shown in Figure 9e. The quantitative and qualitative detection of H_2_S and NH_3_ gases was performed based on the change in color and weight of the adsorbed gases, as indicated by Figure 9f,g.

Considering their extensive pore structure and adsorption sites, MOFs adsorb gas molecules mainly through physical interactions, such as hydrogen bonding, van der Waals forces, and intermolecular forces. According to Table 4, gas sensors utilizing MOFs are primarily operated at room temperature. This preference is attributed to the fact that the adsorption properties typically diminish with rising temperatures, a characteristic of physical adsorption [84]. Therefore, these sensors can effectively operate at room temperature, which allows for simpler structure, lower energy consumption, and better stability than MOS-based sensors. Meanwhile, the abundance of functional groups on MOFs’ surfaces confers them with exceptional gas selectivity. Moreover, the framework and adsorption sites of MOFs are customizable to align with the properties of the target gases, enabling refined detection capabilities [89]. However, the process of physical adsorption is relatively slow, leading to the long response/recovery time of MOF sensors. Moreover, since MOFs are porous framework materials and most of the pore sizes are at the nanometer level, it is challenging for gas molecules with larger molecular weights to penetrate the framework. As shown in Table 4, MOF materials are primarily utilized for inorganic gases (H_2_S, CO_2_, SO_2_, NH_3_) and some short-chain VOCs (methanol, ethanol, acetone, formaldehyde, etc.).

The two preceding sections have detailed two leading gas-sensitive materials, including MOFs and MOS. MOS materials are notably prevalent in resistive sensors. However, MOS’s limited selectivity results in the requirement for a more extensive array of sensors within an e-Nose. Additionally, the need to accommodate various sensors with specific heating devices worsens the complexity of the e-Nose system based on MOS materials. Table 5 presents a summary of the disadvantages and advantages of MOFs and MOSs as gas-sensing materials.

To enhance MOS gas sensor performance, techniques such as doping with precious metals or creating heterojunctions are frequently employed. Despite extensive research and application of these methods, issues with poor selectivity persist. To provide new research insights, this article summarizes the research on MOF materials in the field of gas sensors. These two materials can complement each other in gas sensor performance. Thus, combining these two materials can effectively simplify the structure of the e-Nose device by enhancing the selectivity of the sensor. This paves new avenues for future research.

## 3. Applications of e-Nose

### 3.1. Medical Care

**Figure 10:**
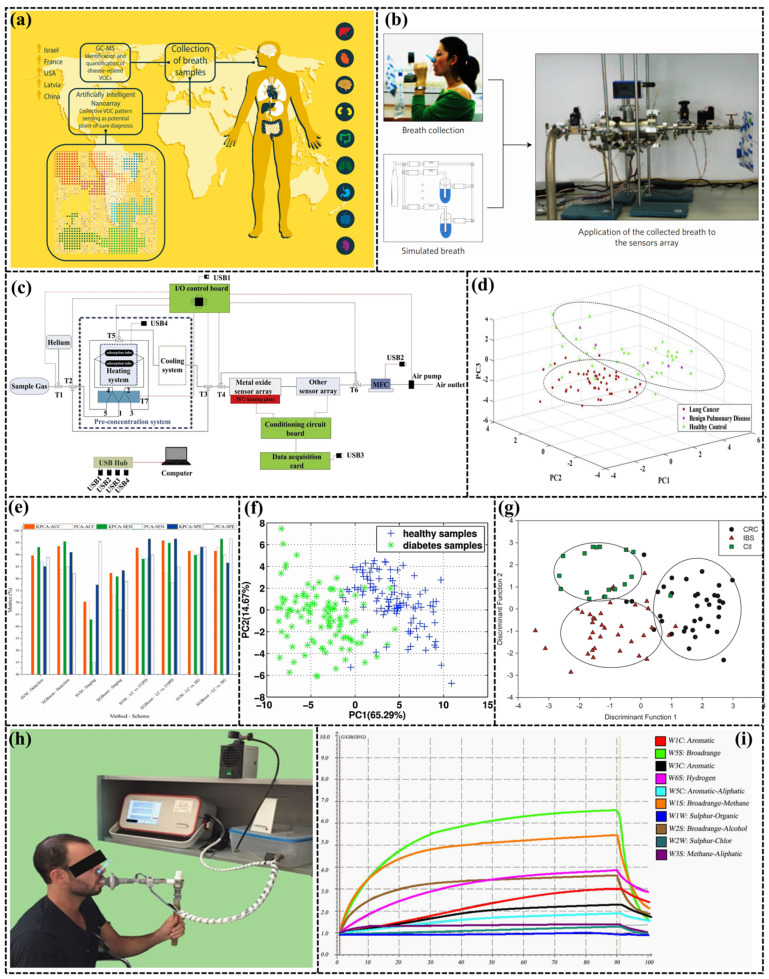
(**a**) Schematic representation of the source of the patients, the types of the diseases and the diagnosis methodology [99]; (**b**) Testing the exhaled breath and simulated breath using the array of gold nanoparticle sensors [101]; (**c**) Schematic diagram of the e-Nose; (**d**) PCA with SGL [112]; (**e**) Results of lung cancer discrimination in four schemes [116]; (**f**) PCA 2-D plot of the sensor signals corresponding to two classes [137]; (**g**) LDA classification separating all three sample groups of CRC, IBS and healthy controls [139]; (**h**) Sampler employed for breath collection; (**i**) Sensor response outputs of a monitored CRC patient [131].

e-Nose research has been increasingly directed towards healthcare applications. Human-exhaled gas includes mainly N_2_, O_2_, CO_2_, and water vapor, with over 500 additional gases present, including acetone, CO, methane, and H_2_, among others, at concentrations in the ppm range. Individual variations in the types and concentrations of VOCs in human breath are common. However, significant deviations in certain hydrocarbons, hydrocarbon derivatives, and benzene derivatives can be used as signals. These changes can be indicative of different health and metabolic conditions [97,98]. The products of cellular metabolism are transported through the circulation to the lungs, where the volatile components are expelled through pulmonary respiration, as shown in Figure 10a [99,100].

Analyzing VOCs in human-exhaled gas is a non-invasive and convenient method for diagnosing diseases, making it a popular alternative for disease diagnosis and early screening. This approach offers several benefits over traditional blood tests and medical imaging techniques, including its non-invasive nature and convenience, as shown in Figure 10b [101,102]. The current application of exhaled-gas detection is mainly divided into ion flow tube mass spectrometry (IFTMS), proton transfer reaction mass spectrometry (PTRMS), gas chromatography–mass spectrometry (GC-MS), and other techniques that can qualitatively and quantitatively analyze trace gases in the exhaled breath. However, these methods are expensive [103,104,105]. With the advantages of miniaturization, low cost, and simple operation, the e-Nose holds a broad application prospect for future application in healthcare. Table 6 summarizes the application of e-Nose in disease diagnosis. Current research has demonstrated that the e-Nose has been applied in the early screening of a variety of diseases, including the detection of diabetes [106,107,108], chronic kidney disease [99,109], lung cancer [110,111,112], and other diseases. For disease detection, especially major diseases such as cancer, diagnostic misinterpretations and incorrect predictions may cause a huge psychological and financial burden to patients. Therefore, disease diagnosis should offer more than just predictive outcomes; it should also convey the confidence level associated with each prediction. This information is crucial for assisting physicians in making the most informed decisions [113].

Lung cancer (LC) is the leading cause of cancer mortality, with more than 1 million deaths worldwide every year. Davide et al. [114] developed an e-Nose consisting of five MOS sensors and tested the e-Nose on lung cancer and at-risk control subjects with a detection accuracy of 77%. While the above report shows excellent results for lung cancer detection, most of the performance does not meet the clinical requirements. According to the medical staff, the accuracy rate of the e-Nose should be over 90% to provide meaningful guidance for doctors to diagnose the disease. To enhance the accuracy, Liu et al. [112] proposed an e-Nose device equipped with a sensor array of 19 sensors and a novel pre-enrichment system. The exhaled breath was analyzed using the sparse group lasso (SGL) feature selection method and feature selection (FS) method, and the results demonstrated an accuracy of 94.25% for lung cancer detection, as shown in Figure 10c,d.

Humidity can significantly affect gas sensors, even up to 90% relative humidity in human-exhaled breath, making an e-Nose necessarily important to accurately test respiratory gas under high-humidity conditions. Andreas et al. [115] doped Pt, Si, Pd, and Ti into SnO_2_ and formed a sensor array that achieved a LOD of 3 ppb for formaldehyde, a lung cancer marker in exhaled breath. This e-Nose was more highly selective for formaldehyde compared to higher acetone, NH3, and ethanol. Chen et al. [116] proposed a self-designed e-Nose system for the noninvasive diagnosis of lung cancer, which could finally achieve an accuracy of 93.59%, by testing 235 breath samples (including 134 healthy people and 101 lung cancer patients), and the KPCA principal component analysis was combined with the eXtreme Gradient Boosting (XGBoost) algorithm. Details are shown in Figure 10e.

Hakim et al. [98] summarized the marker gases in lung cancer, and identified a total of 36 VOCs in seven categories. Conditions such as age, gender, smoking, alcohol consumption, air pollution, and radiation can lead to individual variations in biomarker samples, complicating the process of categorizing these markers [117,118,119].

**Table 6:** Application of e-Nose in disease diagnosis.

Disease	Gas	e-Nose	Sensor Type	Number	Accuracy (%)	Ref.
Diabetes	Acetone, Ethanol, CO, etc.	FIGARO	MOS	12	68.66	[120]
		Self-assembly	MOS	3	100	[108]
		Hanwei	MOS	6	54	[107]
		Self-assembly	MOS	3	-	[121]
		Hanwei	MOS	5	99.44	[106]
		Self-assembly	QCM	8	74.76	[122]
Lung cancer	Formaldehyde, Butane etc.	Self-assembly	MOS, Hot Wire, Solid Electrolyte, Electrochemistry	19	94.25	[112]
		Self-assembly	MOS	5	77	[114]
		Self-assembly	MOS	7	75	[123]
		Self-assembly	MOS (Pt-, Si-, Pd-, Ti-SnO_2_)	4	-	[115]
		Cyranose320	Conducting polymer	32	72	[124]
		Cyranose320	Conducting polymer	32	70	[125]
		Cyranose320	Conducting polymer	32	>80	[126]
		Cyranose320	Conducting polymer	32	93.4	[110]
		Self-assembly	QCM	8	85.7	[111]
		Self-assembly	MOS	11	93.59	[116]
		Self-assembly	QCM	8	85	[127]
Intestinal diseases	H_2_, Methane etc.	Aeonose	MOS	3	84	[128]
		WOLF	Electro-chemical; Infra-red Optical; Photo-ionisation	13	78	[129]
		Cyranose320	Conducting polymer	32	85	[130]
		PEN3	MOS	10	91	[131]
		Self-assembly	MOS	5	95	[132]

In diabetic patients, due to the impaired metabolism of sugar in the body, glucose cannot provide the body with a certain amount of energy; instead, energy is obtained through lipolysis, a process that produces a large amount of acetone, which is eliminated by the flow of blood to the lungs, followed by exhalation [133]. The average concentration of acetone gas in the exhaled gas of adults should be less than 1.9 mg/m^3^, while in the adults with diabetes, it is higher than 4.27 mg/m^3^, and therefore, the concentration of acetone gas in breath is considered a potential biomarker of diabetes [134]. As early as 1997, Wang et al. [135] used an e-Nose device to detect acetone gas by doping SnO_2_ with catalysts such as Sb and Ce. However, most of the gas sensors are cross-sensitive, and it is almost impossible for a single sensor to qualitatively and quantitatively detect the gas. In 2001, Lin et al. [136] used six sensors as a sensing array, and the increase in the number of sensors effectively improved the accuracy of detection for different diseases. Guo et al. [120] adopted an e-Nose consisting of 12 MOS sensors for the detection of diabetic patients, and combined it with the SVOR algorithm. The classification accuracy for diabetic samples can reach 68.66%. In 2012, Guo et al. [137] improved the accuracy to 87% for diabetic patients utilizing an e-Nose consisting of an array of 12 MOS commercial sensors in combination with PCA and KNN methods. Details are shown in Figure 10f.

Tumor growth is accompanied by changes in genes and/or proteins, which may lead to peroxidation of cell membranes, resulting in changes in VOC gases [138]. A variety of VOC gases are produced in the gastrointestinal tract by the complex interactions of colonocytes, human intestinal flora, and invasive pathogens. For example, methanogenic bacteria present in the small intestine convert H_2_ and CO_2_ gases into methane gas, and intestinal diseases affect the imbalance of methanogenic bacteria, leading to changes in the concentration of hydrogen and methane gases in the intestinal tract. Therefore, early screening for rectal cancer and other intestinal diseases can be carried out using an e-Nose [139]. For instance, Westenbrinket et al. [129] utilized the WOLF system (based on an array of 13 commercial electro-chemical and optical sensors) and combined it with the LDA analytical method for detecting colorectal cancer by analyzing the urine odor. Details are presented in Figure 10g.

Furthermore, Meij et al. [130] also employed the commercially available Cyranose320, which houses 32 conductive polymer sensors, to analyze fecal gases for the detection of colorectal cancer. This approach demonstrated a sensitivity of 85% and a specificity of 87%. However, analyzing the odor of either urine or feces can lead to some psychological resistance on the part of the patient, and the sampling and analysis process is cumbersome. Altomare et al. [131] used PEN_3_ to detect rectal cancer by analyzing exhaled gas. Significant improvements are obtained through breath analysis both in terms of psychological acceptance and in the measurement process, as shown in Figure 10h,i.

The breath test outcomes are benchmarked against clinical standards, and the procedure can be concluded in approximately 10 min, significantly enhancing the efficiency of the diagnostic process. However, the application of the e-Nose to the detection of diseases also is still affected by many interfering factors (e.g., age, gender, other diseases, etc.). Hence, augmenting the statistical data in subsequent stages and integrating with existing clinical instruments are essential to refine the test accuracy [132].

### 3.2. Environment Monitoring

For environmental monitoring applications, continuous and real-time detection of target gases is essential. The specific gases targeted for monitoring can vary significantly across different fields of application. For instance, coal-fired power plants are monitored mainly for CO, NO_x_, H_2_S, SO_2_, O_3_, etc. [140,141,142]; air quality is mainly monitored for SO_2_, NO_2_, CO, O_3_, etc. [143,144]; automobile exhaust mainly includes CO, NO_x_, SO_2_, etc. [145]; indoor VOCs include formaldehyde, benzene, toluene, xylene, etc. [146,147]; and soil/water pollution mainly includes toluene, ethylbenzene, trichlorobenzene, methane, NH_3_, SO_2_, etc. [148].

To this end, e-Nose technology has been widely used in environmental monitoring. Notably, one of the pioneering studies on employing an e-Nose for detecting a range of odors in the atmosphere was conducted by Misselbrook in 1997 [149]. Formaldehyde is a common target of indoor air quality testing due to its potential health effects. The reliability of measurement outcomes can be compromised by multiple factors, including humidity, temperature, and the presence of other gases. Additionally, the choice of the correct gas sensing element and the data processing algorithm play crucial roles in ensuring accurate results. Xu et al. [150] analyzed data from the sensing array in the e-Nose using three prediction models, including Back Propagation (BP), Radial Basis Function (RBF), and SVM. In addition, the accuracy of the three methods for detecting indoor formaldehyde concentration was compared, and the results showed that the BP algorithm was more accurate, regardless of whether the weather was heavily or slightly polluted. Dang et al. [151], in an effort to bolster the precision and stability of classification, took into account atmospheric conditions like temperature, humidity, and pressure. They introduced a novel system known as the Improved Support Vector Machine Ensemble (ISVMEN). This system is tailored to address the complexities of multi-class recognition in e-Nose technology, thereby improving the classification of various gas samples. Zhang et al. [152] also compared six classification models (EDC, SFAM, MLP, individual FLDA, single SVM, and the HSVM model) to analyze the e-Nose signals of six gases, including formaldehyde, benzene, toluene, CO, NH_3_, and CO_2_ in indoor air. The results showed that the Hierarchical Support Vector Machine (HSVM) is more suitable for the detection of indoor air pollutants among the six classification methods, and the average accuracy of classification for the six pollutants can reach 93.74% (training–testing proportions: 20–80%).

**Figure 11:**
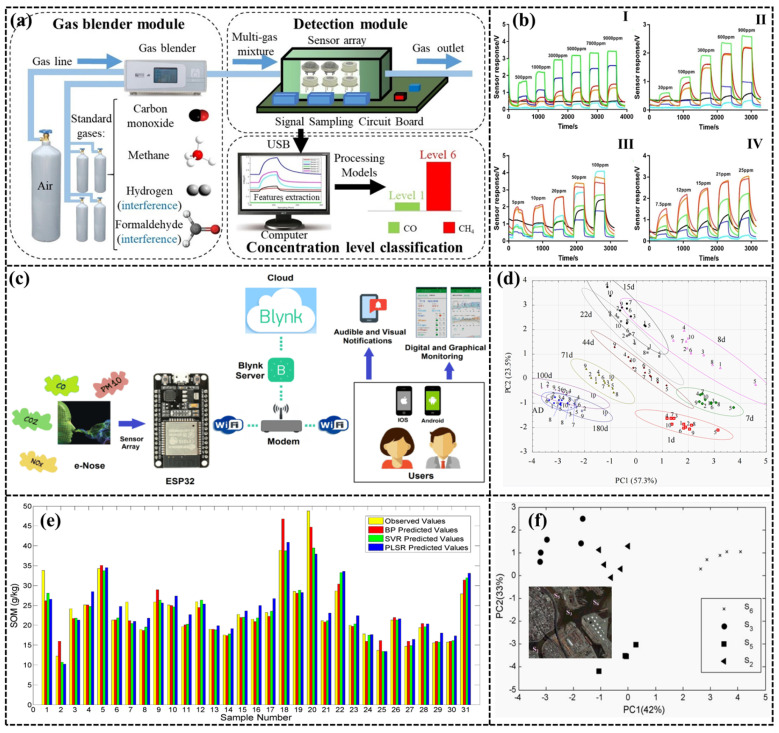
(**a**) The overall diagram of the e-Nose system; (**b**) The voltage-time response of the gas sensor array: (**I**) CH4; (**II**) CO; (**III**) H_2_; (**IV**) CH_2_O [153]; (**c**) The proposed e-Nose system architecture [154]; (**d**) Two-dimensional PCA plots for volatile fingerprints for the 10 studied soils at 10 moistures [157]; (**e**) Comparison of prediction results from different models [159]; (**f**) PCA analysis of the water chemical parameters measured at the different sites [163].

Indoor air pollution not only affects human health but also poses a significant threat to personal safety. For instance, natural gas in the kitchen can cause discomfort, suffocation, and even fire or explosion, thereby directly and seriously harming human health and safety. While natural gas is primarily composed of CO and CH_4_, Zhang et al. [153], by using a sensor array of six MOSs combined with PCA, LDA, and BP-ANN, achieved an accuracy of 93.35% and 93.22% for detecting H_2_ and CH_4_ in the presence of interfering gases H_2_ and formaldehyde, respectively. Figure 11a,b present the test system. Due to the production of IoT-based portable air quality measurement devices and their widespread use, the demand for miniaturized e-Nose devices is increasing for outdoor air quality monitoring. Tastan et al. [154] combined e-Nose with a 32-bit ESP32 Wi-Fi controller and the mobile interface developed by the Blynk IoT platform, and the received data were recorded in a cloud server. The schematic diagram of the testing system is shown in Figure 11c.

There has been limited research on deploying e-Noses for soil and water pollution detection, in contrast to their extensive use in air monitoring. This limitation stems from the diversity of pollutants, the extensive range of contamination levels in water and soil, and the significant environmental condition variability, all of which complicate the accurate acquisition of detection data. Soil pollution has become an urgent environmental problem due to the acceleration of urbanization and the transformation process of legacy sites. Trichloroethylene and perchloroethylene are among the most widespread pollutants in the soil environment [155,156]. Bieganowski et al. [157] used an e-Nose consisting of eight MOS-gas sensors in combination with PCA and ANN methods to categorize soils with different humidity levels and different numbers of days, as depicted in Figure 11d. Lavanya et al. [158] detected hyaluronic acid and free fatty acid content in soil using an e-Nose device consisting of eight MOS-gas sensors. Zhu et al. [159] implemented an e-Nose device consisting of 10 MOS sensors in combination with PLSR, BP-ANN, and SVR algorithms to detect the organic matter content in soil, as shown in Figure 11e.

In water pollution detection, VOCs are partitioned between liquid and gas phases, allowing for their detection in both phases. A gas sensor system that operates continuously can be utilized to monitor the gas phase for these compounds. Therefore, the e-Nose can provide real-time monitoring of wastewater by detecting VOC gas in water, thereby avoiding the disadvantages of complicated testing and long detection time in the traditional liquid-phase detection process [160,161]. Lamagea et al. [162] utilized an e-Nose to monitor the dynamics of pollutants in a river polluted by industrial effluents in the province of Buenos Aires, Argentina, and to analyze and diagnose the environmental quality of the watershed. Goschnick et al. [163] tested wastewater using the commercially available KAMINA e-Nose, and investigated the headspace of water samples polluted with chloroform (as a representative of chloro-organic solvents) or ammonia (representing fecal contamination) under conditions simulating stagnant and flowing waters. Details are shown in Figure 11f. The KAMINA e-Nose serves a variety of applications beyond wastewater detection; it is also utilized for assessing the quality of textiles and evaluating the freshness of meat [164,165]. Table 7 summarizes the application of e-Nose in environment monitoring.

**Table 7:** Application of e-Nose in environmental monitoring.

Application	Gas Detection	Date Analysis Methods	Sensor Number	Accuracy (%)	Ref.
Indoor air monitoring	Formaldehyde, Benzene	BP, PSO	4	-	[166]
Indoor air monitoring	Formaldehyde, Toluene, CO	S4VM	-	84.37–86.92	[167]
Indoor air monitoring	Benzene	LVQ	12	-	[42]
Indoor air monitoring	Toluene, CO, NH_3_	ISVMEN	7	92.58	[151]
Indoor air monitoring	CO_2_, CO, NO_2_	-	4	-	[154]
Indoor air monitoring	Formaldehyde, Benzene, Toluene, CO, NH_3_, CO_2_	EDC, SFAM, MLP-BP, individual FLDA, single SVM	6	93.74	[152]
Indoor air monitoring	Formaldehyde, Benzene, Toluene, CO, NH_3_, NO_2_	LSSVM	4	99.38	[168]
Indoor air monitoring	CO, Methane	PCA, LDA,	6	93.35 (CO); 93.22 (Methane)	[153]
Indoor air monitoring	Formaldehyde	BP, RBF, SVR	4	-	[150]
Vehicle Exhaust	NO_x_	ELM,	-	-	[169]
Ambient air quality	Limonene, Ethanol, Dimethyl sulfide	PCA	6	>85	[170]
Soil	H_2_O	PCA, ANN	8		[157]

### 3.3. Agriculture and Food Safety Monitoring

e-Nose technology is currently extensively used in food safety and agricultural production. It detects VOCs in food, such as formaldehyde, acetone, benzene, and ethanol, to determine the type, ripeness, and quality of food [171]. This non-destructive detection method enables the rapid and precise identification of food odors, spoilage, and additives, facilitating the monitoring and control of food processing [172]. In addition, this method can be used to detect pests and assess crop maturity in agricultural production [173,174].

**Figure 12:**
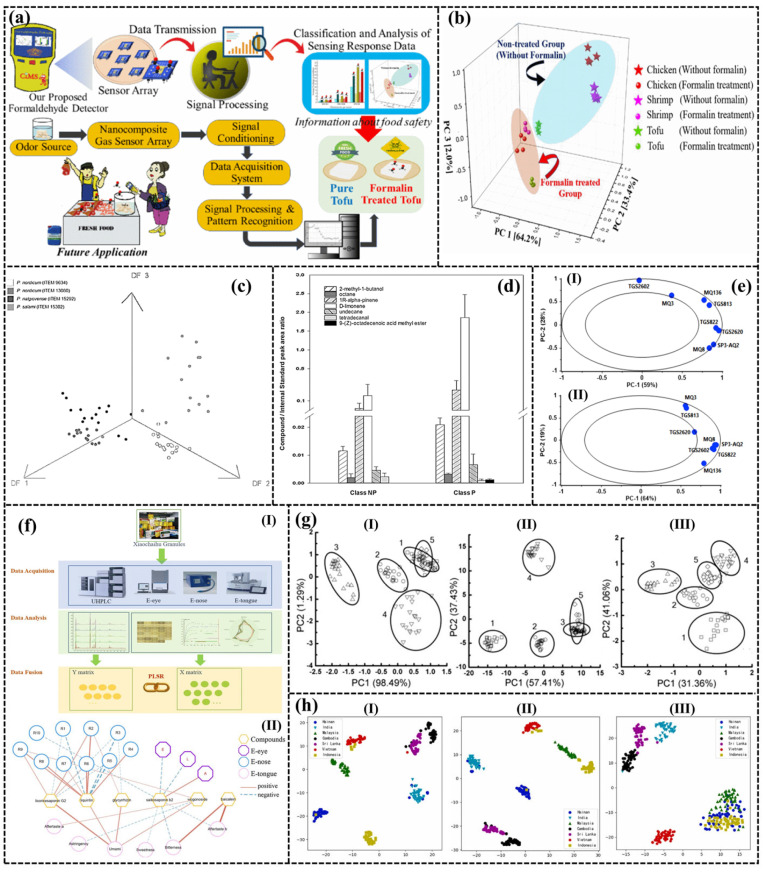
(**a**) e-Nose technology for food safety monitoring; (**b**) Scores plot of a 3D principal component analysis of fabricated sensor array [175]; (**c**) Discriminant Function Analysis (DFA) for meat agar samples; (**d**) Pattern of the volatile compounds showing different contents [185]; (**e**) Loading plot of PCA analysis for adulteration of raw milk with (**I**) hydrogen peroxide and (**II**) sodium hypochlorite [188]; (**f**) Study design for qualitative and quantitative evaluation method of xiaochaihu granules based on electronic eye, e-Nose, electronic tongue and chemometrics ((**I**) System diagram; (**II**) data processing); (**g**) PCA results of five strawberry juices: (**I**) is obtained by e-Nose measurement, (**II**) is obtained by e-Tongue measurement, and (**III**) is obtained by the fusion of e-Nose data and e-Tongue data; (**h**) Feature visualization: (**I**) e-Touch-CNN, (**II**) e-Nose-CNN, (**III**) e-Eye-CNN [198,199,200].

The produced volatile sulfur compounds, hydrocarbons, etc. are usually considered the result of lipolysis and lipid oxidation, and can be used as potential indicator compounds for identifying meat spoilage. Formalin is an aqueous solution of formaldehyde, which is an important disinfectant and antiseptic. However, it is important to note that formaldehyde is corrosive and that its volatile nature can be extremely harmful to the human respiratory and nervous systems. Yu et al. [175] used an e-Nose system to detect the concentration of formaldehyde gas in raw chicken, raw shrimp, and tofu. The purpose was to determine the presence of contamination in these food products, as shown in Figure 12a,b. In addition, the e-Nose can also be used for meat identification [176,177], rapid detection of the type and content of adulterated meat [178], and the traceability of the origin of meat [179,180]. Pulluri et al. [181] conducted qualitative and quantitative detection of counterfeit beef production using an e-Nose technique by adding pork to beef in varying proportions from 0–100%. The data from each sensor were downscaled using the PCA method and then classified using the SVM method. The results demonstrate that the model boasts an accuracy of up to 99.99%, confirming the capability of the e-Nose to precisely analyze and distinguish between genuine and counterfeit beef.

Fruits and vegetables such as peaches, mangoes, and tomatoes are susceptible to fungal contamination during harvesting, processing, transportation, and storage, leading to spoilage of the fruit [182,183]. Currently developed testing methods for measuring fungal contamination include microbial cell counting and enzyme-linked immunosorbent assay. However, these methods are complex and destructive, necessitating the development of a rapid, sensitive, and non-destructive method (such as e-Nose) for on-line and real-time detection of fungi [184]. Fruits produce significant amounts of VOCs during fungal infections, and the concentration and type of volatile gases are usually related to the number and general type of colonies. Not only fruits, but also meats, can produce molds during preservation. For instance, cured meat can produce ochratoxin A and VOCs such as 2-methyl-1-butanol, octane, 1R-α-pinene, D-limonene, undecane, etc. The concentration of such VOC gases is typically proportional to the number of colonies, as shown in Figure 12c,d [185]. Therefore, the quality of fruits can be accurately judged by the e-Nose according to the gas concentration. The e-Nose is also capable of evaluating the quality and freshness of various foods, as well as distinguishing between different types, including diverse parsley and caraway cultivars [186,187].

The e-Nose can also detect liquid food, such as milk [188,189], tea [190,191], and coffee [192,193,194] by identifying the weak differences between odors. It can distinguish between different types of the same food and detect food adulteration, such as different origins of coffee or different varieties of tea [195]. Tohidi et al. [188] proposed an e-Nose device consisting of eight MOS sensors to monitor the degree of fermentation of black tea. Black tea fermentation can be judged not only by the color of the leaves but also by the smell. Therefore, the degree of fermentation of tea was detected by measuring VOC gases such as linalool, nerolidol, benzaldehyde, and phenyl ethanol in the gas, as shown in Figure 12e. Two methods, namely, the 2-Norm method (2NM) and the Mahalanobis distance method (MDM), were tested, and the results were correlated with those of colorimetric tests and human expert evaluation.

Pest detection is crucial for agricultural production to avert the considerable economic losses that pests can inflict each year. However, traditional methods of pesticide and fertilizer application have adverse effects on the ecosystem and human health. Therefore, there is an urgent need to develop more environmentally friendly pest detection technologies [196]. In this case, e-Nose can be used not only for food quality testing, but also for crop health and pest detection [197].

On the one hand, when crops are infested with pests, the plants will release specific gases, and the content and types of gases will change with the types and numbers of pests. Therefore, the e-Nose system can be used to provide timely feedback regarding the damage to crops and effectively reduce losses [173]. For instance, Sun et al. effectively detected tea plants either with different invasive severities or different invasive times by using a MOS-style e-Nose device combined with PCA and MIP data analysis methods.

On the other hand, pests emit specific gases while attacking crops, and the e-Nose can analyze the molecules of these gases to promptly identify the pests. Henderson et al. [174] found that stink bugs in cotton released two specific gases, trans-2-decenal and trans-2-octenal. Both gases were monitored by Cyranose 320 e-Nose. Under laboratory conditions, internal boll injury was predicted 95% of the time and the presence of stink bugs 100% of the time.

Table 8 summarizes the application of the e-Nose to the detection of foodstuffs, including meat, fruits, agricultural products, beverages, etc. The summary includes the characteristic gases, the number of sensors in the e-Nose, and data analysis methods. From the table, for food detection, the characteristic gases are mainly VOCs. The variety of characteristic gases is extensive, necessitating a greater number of gas sensors in the e-Nose. On average, there are about nine sensors used. In sensor data processing, most of the PCA methods are used to downscale the data from each sensor. Then, SVM, PLSR, ANN, and other algorithms are employed to categorize and regress the data according to different needs, confirming the potential of the e-Nose for food and agricultural product detection.

However, the detection process of food is more complicated, involving not only the change in smell, but also the change in color and taste. Therefore, the combination of e-Nose, e-Tongue, and e-Eye technology can greatly increase the detection accuracy, which has also become the new direction of the e-Nose in the subsequent development process. As shown in Figure 12f–h, the combination of e-Eye, e-Nose, and e-Tongue is used to test xiaochaihu granules, strawberry juice, and the origin of black pepper, respectively [198,199,200]. Apetrei et al. [201] combined an e-Nose, an e-Tongue, and an e-Eye for the characterization of olive oils with different degrees of bitterness. After processing the data by PCA and PLS-DA, the polyphenol content of olive oil could be effectively analyzed. Prieto et al. [202] also took a combination of e-Nose, electronic tongue, and electronic eye to analyze the effect of cap type on wine quality.

Currently, the e-Nose is primarily used for crop detection in laboratory settings, with only a few measurements taken in orchards and fields. Detecting VOCs under real environmental conditions presents a challenge, making it necessary to improve the e-Nose’s performance to expand its range of applications [203]. To optimize the e-Nose, subsequent studies must include conditions such as temperature, humidity, and wind speed, considering the effect of environmental fluctuations.

**Table 8:** Agriculture and food safety monitoring.

	Application	Gas	Number	Methods	Ref.
Meat	Detection of formalin in pork	Formaldehyde	3	PCA	[175]
	Prediction of ochratoxin A on dry-cured meat	2-Methyl-1-Butanol, Octane, 1R-α-Pinene, et al.	12	DFA	[185]
	Detection of Meat Spoilage	NH_3_, Amines, Volatile Sulphur compounds etc.	8	PCA, CFWN1111N	[204]
	Quality changes of oxidized chicken fat	2-Alkenal, 2,4-Alkadienal, Carboxylic acid	18	PLSR	[205]
Fruit	Penicillium digitatum in post-harvest oranges	*Penicillium digitatum*	4	PCA	[206]
	Mango quality	Alkenes, Alcohols, Carbon monoxide	8	PCA, SR	[207]
	Peach quality	Terpenes, Aromatic compounds	10	PCA, PLSR	[208]
Agricultural product	Peanut quality	Acid and Peroxide values	10	CA, PCA, PLSR	[209]
	Soft-rot infection in potatoes	CO, NO, Ethylene Oxide	9	PCA	[210]
	Classification of garlic cultivars	Vinyldithiins	8	PCA	[187]
	Quality of cherry tomato	-	14	PCA, ELM, PLSA	[211]
	Caraway cultivars	Aromatic	8	PCA, LDA, SVM	[186]
	Identification of Tobacco Types and Cigarette Brands	Propanone, ethanol, ethyl acetate, and toluene	3	-	[212]
Soft drink	characterize and classify 7 Chinese robusta coffee	-		PCA, KNN, PLSA, BP-ANN	[213]
	Monitoring of black tea	Linalool, Nerolidol, Benzaldehyde, Phenyl ethanol, etc.	8	PCA, 2NM, MDM	[190]
	Aroma profiling of milk adulteration	Formalin, Hydrogen Peroxide, Sodium Hypochlorite	8	PCA, LDA	[188]
	Analysis of the influence of the type of closure in the organoleptic characteristics of a red wine	Phenol	15	PCA, PLS-DA	[202]

## 4. Pattern Recognition and Drift Compensation Algorithms

### 4.1. Pattern Recognition Algorithms

The e-Nose functions as a bionic system, with the sensor array acting as a stand-in for human sensory organs and the pattern recognition algorithm functioning similarly to the human brain, processing and evaluating the final data. The pattern recognition system in the e-Nose is divided into three main parts, including data acquisition and preprocessing, feature selection and extraction, and classification model selection.

#### 4.1.1. Selection and Classification of Features

Feature selection involves choosing the most discriminatory information from the original data, while feature extraction generates a new dataset through mathematical transformations of the original features. The goal of both methods is to select the most effective data features. The purpose of feature selection and extraction is to reduce data redundancy and to improve classification in low dimensions. Jia et al. [214] explained in their article that feature selection in the e-Nose for gas sensors mainly includes maximum response, responses of special time, time of special responses, area (area values of sensor response curve and time axis surrounded), integral, derivative, difference, and second derivative. In their evaluation, Yu et al. [215] analyzed the response curve based on five features, including mean, integral, maximum slope, maximum fluctuation, and maximum value. Furthermore, Marco and Hierlemann et al. [216,217] investigated a range of features, including steady-state response, transient features (such as amplitude and derivative at various points in time), principal component features, phase features, wavelet features, and curve fitting features.

Data extraction can significantly minimize data redundancy, yet the e-Nose’s sensor array continues to produce substantial data volumes, which might potentially diminish the accuracy of subsequent model recognition. Therefore, data dimensionality reduction can enhance the recognition rate and increase the algorithm’s processing speed. Nonlinear techniques can be further divided into kernel function-based and eigenvalue-based methods. Techniques for reducing dimensionality can be categorized as linear or nonlinear. The table summarizes that e-Noses mainly use PCA, a linear, unsupervised pattern recognition method for data analysis, classification, and dimensionality reduction. In the e-Nose, PCA is primarily used to eliminate redundant data from sensor signals [218].

LDA is widely employed for dimensionality reduction, which projects high-dimensional data into a space that best discriminates between classes. PCA is unsupervised and seeks to maximize variance. Conversely, LDA is a supervised algorithm that focuses on class separability. PCA projects the high-dimensional data into a low-dimensional space with as little information loss as possible for data simplification [219,220]. Abdullah et al. identified odor information in diabetic wounds using the LDA method. Izadi et al. [186] used an e-Nose combined with LDA to identify export caraway cultivars. Tohidi et al. [188] proposed a combined algorithm using PCA + LDA for aroma profiling to detect milk adulteration.

#### 4.1.2. Classification Model Selection

When extracting multiple features, the optimal subset should be necessarily selected using a feature selection algorithm. If the analysis purpose is to determine the type of gas or some discrete attribute of the measured substance (such as disease or health), the extracted features can be classified. Commonly used classifiers in the study of e-Nose include KNN, ANN, SVM, and LR [213]. If the analysis aims to evaluate the concentration of an odor or a continuous attribute value of a measured substance (such as blood glucose level), common e-Nose algorithms used include PLSA, ANN, and SVM [221]. Different pattern recognition algorithms have their own advantages and disadvantages when processing e-Nose data. For instance, LR is a classification model that is straightforward to understand and implement. However, its potential drawback is that it may not achieve high classification accuracy. Algorithms such as KNN and LDA do not require repeated sample training, and their models are relatively simple. Nevertheless, their robustness is not strong [186]. The SVM algorithm achieves high classification accuracy in small sample sizes, but its performance decreases when the number of feature dimensions is much larger than that of samples. On the other hand, the ANN algorithm offers greater fault tolerance but demands considerable hardware resources and time for model construction. Consequently, designers can choose the appropriate algorithm based on specific needs and the characteristics of the data at hand [213].

### 4.2. Drift Compensation Algorithm

Signal drift presents a significant challenge for the e-Nose in practical applications. Currently, instrumental variation and time-varying drift are the major factors affecting the e-Nose. Due to the limitations of current technology, signal drift remains an unavoidable problem for the e-Nose [12]. Therefore, an effective sensor drift compensation method is proposed to improve the accuracy and generalization of the e-Nose. From the perspective of machine learning and migration learning, there are three types of correction methods, including component correction methods, adaptive correction methods, and migration learning methods [112].

Regarding component-based calibration, Haugen et al. [222] used a drift compensation algorithm based on the fitting of time-varying curves of calibration sample sensor signals. This procedure eliminates sensor drift in single and multiple measurement sequences (days, months). Fonollosa et al. [11] developed a calibration model using data collected by the master unit and directly normalized the mapping of the signals from the slave units to the space of the master unit during calibration, reducing the cost of calibration. Ziyatdinov et al. [223] suggested using common component principal analysis to determine the direction of variance followed by all gases in the feature space. This new method, eliminating the need for a reference gas, performs equally well as the conventional approach that relies on a best-fit reference gas.

In terms of adaptive calibration, adaptive correction methods refer to algorithms that adaptively match the current sensor output or change the model of the pattern recognition algorithm to achieve drift suppression. Carlo et al. [224] proposed a method based on the Covariance Matrix Adaptation Evolution Strategy (CMA-ES) for stochastic optimization of complex problems. Martinelli et al. [225] proposed an Artificial Immune Network (AINET) algorithm inspired by the natural immune system. This algorithm allows for classification models mostly unaffected by sensor drift. While Adaptive Resonance Theory and the Self Organizing Map exhibit some degree of drift suppression, their ability to offset drift heavily relies on the statistics of class occurrence. This reliance can lead to failure in cases of class presentation imbalance [225].

For transfer learning, migration learning methods aim to suppress drift by adjusting data distribution. These methods are typically categorized as either feature-based or model-based. Specifically, Yan et al. [226] proposed a Drift-Corrected Autoencoder (DCAE) for feature-based methods, enhancing the robustness of the e-Nose system and significantly improving its performance in real-world applications. Liu et al. [227] introduced a unified subspace migration framework known as Cross-domain Extreme Learning Machine (CdELM) for inter-board variance correction in model-based approaches. For model-based approaches, Vergara et al. [228] proposed an integrated SVM-based approach using a weighted combination of classifiers trained at different time points. The ensemble of classifiers effectively handles sensor drift, but a significant number of labeled samples are required. Vito et al. [229] found that semi-supervised learning maintains an excellent drift counteraction effect despite a low number of labeled samples. Martinelli et al. proposed a data processing architecture based on a set of sub-classifiers. In real datasets, the classification rate achieved by the model largely outperforms that of KNN. Meanwhile, Yan proposed two migrated-sample based learning methods for correcting the drift of an e-Nose applied to diabetes detection.

In summary, migration learning methods offer several benefits, such as eliminating the need for direct modeling of intricate data offset patterns. As a result, employing these methods to address sensor drift or variance between plates is gaining popularity.

## 5. Conclusions

e-Nose technology is emerging as a novel and promising technology for the quantitative and qualitative detection of complex gases in practical applications. It is mainly composed of sensor arrays and pattern recognition systems, and the classification accuracy of target gases can be greatly improved by selecting appropriate sensors and pattern recognition algorithms according to different application conditions. In this review, the e-Nose is hereby reviewed based on diverse types of materials such as traditional MOS materials and porous nanomaterials (MOFs), and the applications of the e-Nose are summarized in fields including disease diagnosis, environmental monitoring, and agriculture/food safety monitoring. Finally, pattern recognition and drift compensation algorithms commonly used in the e-Nose are summarized based on practical applications.

On sensor arrays, MOS-type gas sensors are commonly used in the research of the e-Nose, and this review focuses on the application of ZnO, SnO_2_, and Fe_2_O_3_ gas sensors. The sensing mechanism of MOS-type gas sensors operates by reacting adsorbed oxygen on the material’s surface with the target gas. This interaction leads to a change in the material’s resistance value, which correlates with the concentration of the target gas. MOS-type gas sensors are commonly used, and their performance can be improved by constructing heterojunctions, doping with metals, UV irradiation, and adjusting microscopic morphology. However, these sensors still face challenges of poor selectivity and humidity instability. MOFs are a new class of porous materials that offer several advantages, including excellent selectivity, the ability to tailor and optimize materials for specific target gases, and the capability to operate at low detection temperatures. However, they do have drawbacks, such as long response and recovery times. Therefore, future research could explore the combination of MOFs with MOS sensors to leverage the strengths of both, compensating for their individual weaknesses and enhancing overall performance.

The e-Nose can detect a wide range of diseases, including diabetes, cancer, and intestinal diseases, making it useful in healthcare applications. To provide meaningful guidance for disease diagnosis and improve decision-making, it is crucial for the e-Nose to be highly accurate in disease detection. In environment monitoring, the e-Nose is primarily utilized for detecting indoor air quality and combustible gases in stable conditions, with the accuracy of results directly impacting health. Common target gases include formaldehyde, benzene, toluene, methane, and CO. In agriculture and food safety monitoring, selecting the appropriate algorithm to reduce interference is crucial due to the complexity of the detection conditions. PCA is one of the commonly used methods for dimensionality reduction, while SVM, PLSR, and ANN are popular classification methods.

Collectively, most of the current research on the e-Nose remains at the laboratory stage, and detecting target gases under real-world environmental conditions continues to pose significant challenges. Temperature, humidity, and the presence of interfering gases continue to be significant challenges affecting the accuracy of the electronic nose and sensor measurements in ongoing research. Consequently, the research on e-Noses is gradually evolving towards the development of practical applications. For instance, a recent study conducted by Tsinghua University proposed a biomimetic olfactory strategy for gas sampling in electronic noses. This strategy innovatively employs multiple overlapping sniffs to generate dynamic and rich temporal signals in the sensor array of electronic noses. Hence, the perception and recognition ability of different gases are considerably enhanced [230]. To address the issue of a high number of sensors in electronic noses, Yi et al. developed a multivariable sensor that boasts the advantages of a compact design and affordability. Its ability to produce multiple signals that are either partially or completely independent makes it an ideal alternative to traditional sensor arrays [231].

## Figures and Tables

**Table 5:** Advantages and disadvantage of MOFs and MOS as gas materials.

q	Advantage	Disadvantage
MOFs	Selectively adsorb the target gas at room temperature and adjust the adsorption sites according to the target gas; mainly uses capacitive gas sensors, resulting in lower energy consumption; the detection limit is relatively low.	Due to physical adsorption, the response/recovery time is relatively long; usually in powder form, it is not conducive to direct application in electronic devices.
MOS	High degree of responsiveness, stability, and maturity in its preparation process.	MOS gas sensors are predominantly resistive and necessitate heating conditions, resulting in high energy consumption. Furthermore, the absence of specific adsorption sites leads to a lack of selectivity.

## Data Availability

This article contains no data or material other than the articles used for the review and referenced.
